# Supplementary material for: Folate Repletion after Deficiency Induces Irreversible Genomic and Transcriptional Changes in Human Papillomavirus Type 16 (HPV16)-Immortalized Human Keratinocytes
Source: Int J Mol Sci. 2019 Mar 4;20(5):1100. doi: 10.3390/ijms20051100 (PMC6429418; doi:10.3390/ijms20051100)
Supplement: Supplementary file 1 [file ijms-20-01100-s001.pdf]

International Journal of *Molecular Sciences*

```
                                 Ecl136II                   SmaI
AACCGTCAGATCCGCTAGCGCTACCGGACTCAGATCTCGAG                   GGGATCCACCGGTCGCCACCAT
TTGGCAGTCTAGGCGATCGCGATGGCCTGAGTCTAGAGCTC                   CCCTAGGTGGCCAGCGGTGGTA
                                                                                     Loss      Misincorporation

FC
AACCGTCAGATCCGCTAGCGCTACCGGACTCAGATCTCGAG                     GATCCACCGGTCGCCACCAT   0/-2
AACCGTCAGATCCGCTAGCGCTACCGGACTCAGATCTCGAG                   GGGATCCACCGGTCGCCACCAT   0/0
AACCGTCAGATCCGCTAGCGCTACCGGACTCAGATCTCGAG                   GGGATCCACCGGTCGCCACCAT   0/0
AACCGTCAGATCCGCTAGCGCCACCGG                                             TCGCCACCAT   -14/-12
AACCGTCAGATCCGCTAGCGCTACCGGACTCAGATCTCGAG                    GGATCCACCGGTCGCCACCAT   0/-1
AACCGTCAGATCCGCTAGCGCTACCGGACTCAGATCTCGAG                   GGGATCCACCGGTCGCCACCAT   0/0
AACCGTCAGATCCGCTAGCGCTACCGGACTCAGATCTCGAG                   GGGATCCACCGGTCGCCACCAT   0/0
AACCGTCAGATCCGCTAGCGCTACCGGACTCGGATCCACCG                              GTCGCCACCAT   0/-11              4
AACCGTCAGATCCGCTAGCGCTACCGGACTCAGATCTCGAG                       TCCACCGGTCGCCACCAT   0/-4               2
AACCGTCAGATCCGCTAGCGCTACCGGACTCAGATCTCGAG                   GGGATCCACCGGTCGCCACCAT   0/0
AACCGTCAGATCCGCTAGCGCTACCGGACTCAGATCTCGAG                   GGGATCCACCGGTCGCCACCAT   0/0
AACCGTCAGATCCGCTAGCGCTANNGGACTCAGATCTCGAG                   GGGATCCACCGGTCGCCACCAT   0/0
AACCGTCAGATCCGCTAGCGCTACCGGACTCAGATCTCGAG                   GGGATCCACCGGTCGCCACCAT   0/0
AACCGTCAGATCCGCTAGCGCTACCGGACTCAGATCTCGAG                   GGGATCCACCGGTCGCCACCAT   0/0
AACCGTCAGATCCGCTAGCGCTACCGGACTCAGAT                                    GTCGCCACCAT   -6/-11

FD
AACCGTCAGATCCGCTAGCGCTACCGGACTCAGATCTCGAG                   GGGATCCACCGGTCGCCACCAT   0/0
AACCGTCAGATCCGCTAGCGCTACCGGACTCAGATCTCGA                              GGTCGCCACCAT   -1/-10
AACCGTCAGATCCGCTAGCGCTA     CTCAGATCTC                                               -8/-35
AACCGTCAGATCCGCTAGCGCTACCGGACTCAGATCTC                    AAGGGATCCACCGGTCGCCACCAT   -3/0               2
AACCGTCAGATCCGCTAGCGCTACCGGACTCAGATCTCGAG                   GGGATCCACCGGTCGCCACCAT   0/0
AACCGTCAGATCCGCTAGCGCTACCGGACTCAGATCTCGAG                   GGGATCCACCGGTCGCCACCAT   0/0
AACCGTCAGATCCGCTAGCGCTACCGG                                             TCGCCACCAT   -14/-12
AACCGTCAGATCCGCTAGCGCTACCGGACTCAGATCTCGAG                   GGGATCCACCGGTCGCCACCAT   0/0
AACCGTCAGATCCGCTAGCGCTACCGGACTCAGATCTCGAG                       TCCACCGGTCGCCACCAT   0/-4
AACCGTCAGATCCGCTAGCGCTACCGGACTCAGATCTCG                      GGATCCACCGGTCGCCACCAT   -2/-1
GGC                                                                           GAGG   -38/-31            2
AACCGTCAGATCCGCTAGCGCTACCGGACTCAGATCTCGAG                   GGGATCCACCGGTCGCCACCAT   0/0
AACCGTCAGATCCGCTAGCGCTACCGGACTCAGATCTCGAG                   GGGATCCACCGGTCGCCACCAT   0/0
AACCGTCAGATCCGCTAGCGCTACCGGGATC                                   CACCGGTCGCCACCAT   -10/-6
AACCGTCAGATCCGCTAGCGCTACCGGACTCAGATCTCGAG                   GGGATCCACCGGTCGCCACCAT   0/0

FR
AACCGTCAGATCCGCTAGCGCTACCGGACTCAGATCTCGAG                   GGGATCCACCGGTCGCCACCAT   0/0
AACCGTCAGATCC                                                      ACCGGTCGCCACCAT   -25/-7
AACCGTCAGATCCGCTAGCG  A  GGACTCAGATCTCGAG                   GGGATCCACCGGTCGCCACCAT   4/0
AACCGTCAGATCCGCTAGCGCTACCGGACTCAGATCTCGAG                   GGGATCCACCGGTCGCCACCAT   0/0
AACCGTCAGATCCGCTAGCGCTACCGGACTCAGATCTCGAG                   GGGATCCACCGGTCGCCACCAT   0/0
AACCGTCAGATCCGCTAGC                                                              T   -22/-21
AACCGTCAGATCCGCTAGCGCTACCGGACTCAGATCTCGAG                    GGATCCACCGGTCGCCACCAT   0/-1
AACCGTCAGATCCGCTAGCGCTACCGGACTCAGATCTCGAG                   GGGATCCACCGGTCGCCACCAT   0/0
AACCGTCAGATCCGCTAGCGCTAGGGCGAGGAGCTGTTCAC     GGGCGAGGAGCTGTTCACCGGGGTGGT GC  CCAT   0/0                39
AACCGTCAGATCCGCTAGCGCTACCGGACTCAGATCTCGAG                   GGGATCCACCGGTCGCCACCAT   0/0
AACCGTCAGATCCGCTAGCGCTACCGGACTCAGATCTCGAG                   GGGATCCACCGGTCGCCACCAT   0/0
AACCG                                                                  GTCGCCACCAT   -33/-11
AACCGTCAGGTCCGCTAGCGCTACCGGACTCAGATCTCGAG                   GGGATCCACCGGTCGCCACCAT   0/0
AACCGTCAGATCCGCTAGCGCTACCGGACTCAGATCTCGAG                   GGGATCCACCGGTCGCCACCAT   0/0
AACCGTCAGATCCGCTAGCGCtaCCGGACTCAGATCTCG                     GGGATCCACCGGTCGCCACCAT   -2/0
```

Figure S1. Fidelity of DNA damage repair by NHEJ In vivo DNA-ligation assay on FC, FD, and FR cells after transfecting linearized plasmids. Sequence stretches of at the blunt-ended junction. The right columns indicate the number of nucleotide losses and misincorporations, resulting from two independent experiments with minimum seven sequenced clones.

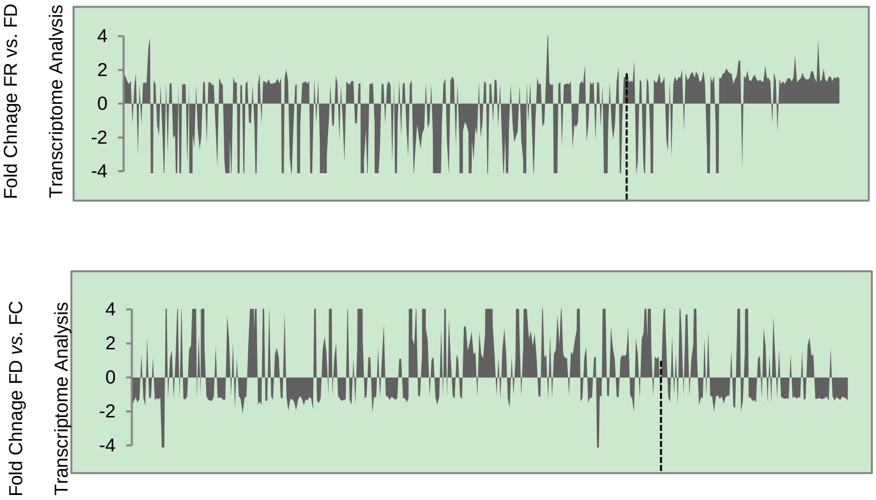

Figure S2 Transcript profile of chromosome 8 genes (**A**) FR vs. FD; (**B**) FD vs. FC. The ordinate expresses the fold changes of genes on chromosome 8.

Table S1. Overview of the primers used for qPCR.

Primers qPCR	Sequence 5´- -3´
CDKN1C Fw	CAGTGGACCGAAGTGGACA
CDKN1C Rv	GGGACCGGGACACTAGGC
H2AFY2 Fw	TCCACTGTCACATCCCTCAG
H2AFY2 Rv	ATCAAAGTGGGCTGAGATGG
LEF1 Fw	GACGAGATGATCCCCTTCAA
LEF1 Rv	AGGGCTCCTGAGAGGTTTGT
DIRAS3 Fw	TGCCGACCATTGAAAATACC
DIRAS3 Rv	CAGGGTTTCCTTCTTGGTGA
SORBS2 Fw	AAGCACAGCCTGCAAGACCA
SORBS2 Rv	TGGGGTATTGGAGGGTCAGG
SLFN11 Fw	GCCCGATAACCTTCACACTC
SLFN11 Rv	ACCCATTTCTCGGTTGTCAG
DCN Fw	GGACCGTTTCAACAGAGAGG
DCN Rv	TCAGAACACTGGACCACTCG
RUNX3 Fw	ACAGCCCCAACTTCCTCTG
RUNX3 Rv	GCTCAGCGGAGTAGTTCTCG
TBX18 Fw	GCGAAAAGGGTTCTTCTGAG
TBX18 Rv	GTGACGCCAGAGGGGAAG
HSPB7 Fw	AGAAGGCCCTGAGCATGTTT
HSPB7 Rv	TGAGAAGTCTCTCACGTCCAC
GALC Fw	GCCAAGCGTTACCATGATTT
GALC Rv	GCAGAGATGGACTCCCAGAG
USP44 Fw	TCCCCACTTCTCAAAGGAAA
USP44 Rv	CATGGCTGGGAAGCAATATC
FAP Fw	TTTTGGCATATGCGGAATTT
FAP Rv	ACGCAGGGTAAGTGGTATCG
KLK5 Fw	AAGGCCCAACCAGCTCTACT

KLK5 Rv	ATTTGACCCCCTGGAACATC
KLK10 Fw	TGTCCTGGTGGACCAGAGTT
KLK10 Rv	TGGTGGTACTTGGGATGGAC
PIM1 Fw	GCTCGGTCTACTCAGGCATC
PIM1 Rv	CCAGTCCAGGAGCCTAATGA
GAPDH Fw	GGAGCGAGATCCCTCCAAAAT
GAPDH Rv	GGCTGTTGTCATACTTCTCATGG
β-ACTIN Fw	CATGTACGTTGCTATCCAGGC
β-ACTIN Rv	CTCCTTAATGTCACGCACGAT

International Journal of Molecular Sciences

MDPI

Table S2. Unique SVs in protein coding genes found in FR cells. SVs that affect exonic sequences are shown in bold and cancer related genes are marked in grey.

Chrom 1	start1	end1	Chrom 2	start2	end2	svtype	Event Score	eventSize	gene1	cancerGene1	gene2	cancerGene2	directFusionCandidates	Fragile sites
**1**	**12708095**	**12708096**	**1**	**12711878**	**12711879**	**DUP**	**5**	**3783**	**AADACL4_intron2**	**.**	**AADACL4_intron3**	**.**	**AADACL4_intron2-AADACL4_intron3**	
**1**	**12708374**	**12708375**	**1**	**12712048**	**12712049**	**DEL**	**5**	**3674**	**AADACL4_intron2**	**.**	**AADACL4_intron3**	**.**	**AADACL4_intron2-AADACL4_intron3**	
1	32389990	32389991	1	32390011	32390012	DUP	5	21	PTP4A2_intron8	.	PTP4A2_intron8	.	.	
**1**	**40034613**	**40034614**	**1**	**40035265**	**40035266**	**DEL**	**3**	**652**	**PABPC4_intron6**	**.**	**PABPC4_6**	**.**	**PABPC4_intron6-PABPC4_6**	
**1**	**212459633**	**212459634**	**1**	**212502474**	**212502475**	**DEL**	**5**	**42841**	**PPP2R5A_intron2**	**PPP2R5A**	**PPP2R5A_intron6**	**PPP2R5A**	**PPP2R5A_intron2-PPP2R5A_intron6**	
**1**	**212530021**	**212530022**	**1**	**212530289**	**212530290**	**DEL**	**5**	**268**	**PPP2R5A_intron15**	**PPP2R5A**	**PPP2R5A_intron15**	**PPP2R5A**	**.**	
**1**	**212530398**	**212530399**	**1**	**212530496**	**212530497**	**DEL**	**3**	**98**	**PPP2R5A_15**	**PPP2R5A**	**PPP2R5A_intron16**	**PPP2R5A**	**PPP2R5A_15-PPP2R5A_intron16**	
2	8236260	8236261	2	8236281	8236282	DUP	5	21	AC007464.1_intron1, LINC00299_intron15	.	LINC00299_intron15, AC007464.1_intron1	.	.	
2	12872146	12872147	2	12872126	12872127	DUP	5	20	TRIB2_intron4	.	TRIB2_intron4	.	.	
2	36605475	36605476	2	36605495	36605496	DUP	5	20	CRIM1_intron3	.	CRIM1_intron3	.	.	
2	59610792	59610793	2	59610842	59610843	DEL	5	50	RP11-444A22.1_intron3, AC007131.2_intron8	.	RP11-444A22.1_intron3, AC007131.2_intron8	.	.	

**2**	**141566325**	**141566326**	**2**	**142182126**	**142182127**	**DEL**	**5**	**615801**	**LRP1B_intron33**	**LRP1B**	**LRP1B_intron4**	**LRP1B**	**LRP1B_intron33-LRP1B_intron4**	***FRA2F***
**2**	**141648306**	**141648307**	**2**	**142127053**	**142127054**	**DEL**	**5**	**478747**	**LRP1B_intron24**	**LRP1B**	**LRP1B_intron4**	**LRP1B**	**LRP1B_intron24-LRP1B_intron4**	***FRA2F***
**2**	**141913468**	**141913469**	**2**	**142018832**	**142018833**	**DEL**	**5**	**105364**	**LRP1B_intron8**	**LRP1B**	**LRP1B_intron4**	**LRP1B**	**LRP1B_intron8-LRP1B_intron4**	***FRA2F***
2	200661296	200661297	2	200661334	200661335	DEL	5	38	FTCDNL1_intron6	.	FTCDNL1_intron6	.	.	
2	203155429	203155430	2	203155481	203155482	DUP	5	52	NOP58_intron9	.	NOP58_intron9	.	.	*FRA2I*
2	240694378	240694379	2	240694400	240694401	DUP	5	22	AC093802.1_intron2	.	AC093802.1_intron2	.	.	*FRA2J*
3	37101746	37101747	3	37101768	37101769	DUP	5	22	LRRFIP2_intron32, MLH1_intron24	MLH1	MLH1_intron24, LRRFIP2_intron32	MLH1	.	
**Chrom 1**	**start1**	**end1**	**Chrom 2**	**start2**	**end2**	**svtype**	**Event Score**	**eventSize**	**gene1**	**cancerGene1**	**gene2**	**cancerGene2**	**directFusionCandidates**	**Fragile sites**
**3**	**50330752**	**50330753**	**3**	**50331420**	**50331421**	**INV**	**5**	**668**	**HYAL3_6**	**.**	**HYAL3_intron4**	**.**	**HYAL3_6-HYAL3_intron4**	
**3**	**50330976**	**50330977**	**3**	**50332127**	**50332128**	**INV**	**5**	**1151**	**HYAL3_intron5**	**.**	**HYAL3_intron4**	**.**	**HYAL3_intron5-HYAL3_intron4**	
**3**	**58417357**	**58417358**	**3**	**58417601**	**58417602**	**DEL**	**5**	**244**	**PDHB_1**	**PDHB**	**PDHB_1**	**PDHB**	**.**	
**3**	**60384818**	**60384819**	**3**	**60578611**	**60578612**	**DEL**	**5**	**193793**	**FHIT_intron9**	**FHIT**	**FHIT_intron8**	**FHIT**	**FHIT_intron9-FHIT_intron8**	***FRA3B***
3	60846107	60846108	3	60851397	60851398	DEL	5	5290	FHIT_intron6	FHIT	FHIT_intron6	FHIT	.	*FRA3B*
3	60957459	60957460	3	60991335	60991336	DEL	5	33876	FHIT_intron6	FHIT	FHIT_intron6	FHIT	.	*FRA3B*
**3**	**64641346**	**64641347**	**3**	**64641409**	**64641410**	**DEL**	**5**	**63**	**ADAMTS9_5**	**ADAMTS9**	**ADAMTS9_5**	**ADAMTS9**	**.**	
3	131898759	131898760	3	131898779	131898780	DUP	5	20	CPNE4_intron2	.	CPNE4_intron2	.	.	
3	134890706	134890707	3	134890726	134890727	DUP	5	20	EPHB1_intron23	EPHB1	EPHB1_intron23	EPHB1	.	

3	135994735	135994736	3	136002097	136002098	DEL	5	7362	PCCB_intron11	.	PCCB_intron11	.	.	
3	141211928	141211929	3	141211908	141211909	DUP	5	20	RASA2_intron2	RASA2	RASA2_intron2	RASA2	.	
**3**	**151151519**	**151151520**	**3**	**151151567**	**151151568**	**DEL**	**5**	**48**	**MED12L_48,IGSF10_7**	**IGSF10,MED12L**	**MED12L_48,IGSF10_7**	**IGSF10,MED12L**	**.**	***FRA3D***
4	8039292	8039293	4	8039317	8039318	DUP	5	25	ABLIM2_intron11	ABLIM2	ABLIM2_intron11	ABLIM2	.	*FRA4A*
4	68609981	68609982	4	68610001	68610002	DUP	5	20	GNRHR_intron2	.	GNRHR_intron2	.	.	
4	77146749	77146750	4	77147058	77147059	DEL	5	309	FAM47E_intron3	.	FAM47E_intron3	.	.	
4	79858845	79858846	4	79858825	79858826	DUP	5	20	PAQR3_intron1	.	PAQR3_intron1	.	.	
**4**	**144459865**	**144459866**	**4**	**144459938**	**144459939**	**DEL**	**5**	**73**	**SMARCA5_intron13**	**SMARCA5**	**SMARCA5_13**	**SMARCA5**	**SMARCA5_intron13-SMARCA5_13**	
**4**	**144466001**	**144466002**	**4**	**144466625**	**144466626**	**DEL**	**5**	**624**	**SMARCA5_intron18**	**SMARCA5**	**SMARCA5_18**	**SMARCA5**	**SMARCA5_intron18-SMARCA5_18**	
**4**	**144468056**	**144468057**	**4**	**144468534**	**144468535**	**DEL**	**5**	**478**	**SMARCA5_20**	**SMARCA5**	**SMARCA5_intron21**	**SMARCA5**	**SMARCA5_20-SMARCA5_intron21**	
4	150639707	150639708	4	150639885	150639886	DEL	5	178	RP11-526A4.1_intron10	.	RP11-526A4.1_intron10	.	.	
4	170378280	170378281	4	170378366	170378367	DEL	5	86	NEK1_intron26	.	NEK1_intron26	.	.	
5	21834222	21834223	5	21837527	21837528	DEL	5	3305	CDH12_intron14	CDH12	CDH12_intron14	CDH12	.	*FRA5E*
5	53366200	53366201	5	53366662	53366663	DUP	3	462	ARL15_intron6	.	ARL15_intron6	.	.	
**5**	**65111984**	**65111985**	**5**	**65316190**	**65316191**	**DUP**	**5**	**204206**	**NLN_intron15**	**NLN**	**ERBB2IP_intron9**	**ERBB2IP**	**NLN-ERBB2IP**	
**Chrom 1**	**start1**	**end1**	**Chrom 2**	**start2**	**end2**	**svtype**	**Event Score**	**eventSize**	**gene1**	**cancerGene1**	**gene2**	**cancerGene2**	**directFusionCandidates**	**Fragile sites**
**5**	**68470236**	**68470237**	**5**	**68470708**	**68470709**	**DEL**	**5**	**472**	**CCNB1_intron6**	**CCNB1**	**CCNB1_6**	**CCNB1**	**CCNB1_intron6-CCNB1_6**	
5	96086180	96086181	5	96086200	96086201	DUP	5	20	CAST_intron33	.	CAST_intron33	.	.	*FRA5D*
5	138699739	138699740	5	138699818	138699819	DUP	3	79	PAIP2_intron4	.	PAIP2_intron4	.	.	

5	141051009	141051010	5	141051029	141051030	DUP	5	20	ARAP3_intron12	ARAP3	ARAP3_intron12	ARAP3	.	
5	161020192	161020193	5	161020250	161020251	DUP	5	58	GABRA6_intron2	GABRA6	GABRA6_intron2	GABRA6	.	
5	175096921	175096922	5	175096941	175096942	DUP	5	20	HRH2_intron2	.	HRH2_intron2	.	.	
6	6157205	6157206	6	6160593	6160594	DEL	5	3388	F13A1_intron15	F13A1	F13A1_intron15	F13A1	.	*FRA6B*
6	42103048	42103049	6	42104632	42104633	DEL	5	1584	C6orf132_intron1	.	C6orf132_intron1	.	.	
6	69731019	69731020	6	69731040	69731041	DUP	5	21	BAI3_intron14	BAI3	BAI3_intron14	BAI3	.	
6	79767649	79767650	6	79767669	79767670	DEL	5	20	PHIP_intron6	PHIP	PHIP_intron6	PHIP	.	
6	109790036	109790037	6	109790094	109790095	DEL	5	58	ZBTB24_intron5	.	ZBTB24_intron5	.	.	
**6**	**111213647**	**111213648**	**6**	**111213937**	**111213938**	**DEL**	**3**	**290**	**AMD1_intron8**	**.**	**AMD1_8**	**.**	**AMD1_intron8-AMD1_8**	***FRA6F***
6	112086339	112086340	6	112086361	112086362	DUP	5	22	FYN_intron11	FYN	FYN_intron11	FYN	.	*FRA6F*
6	149907785	149907786	6	149907765	149907766	DUP	5	20	GINM1_intron9	.	GINM1_intron9	.	.	
**6**	**162203926**	**162203927**	**6**	**162333168**	**162333169**	**DUP**	**5**	**129242**	**PARK2_intron7**	**.**	**PARK2_intron6**	**.**	**PARK2_intron7-PARK2_intron6**	***FRA6E***
6	162492756	162492757	6	162492947	162492948	DEL	5	191	PARK2_intron4	.	PARK2_intron4	.	.	*FRA6E*
**7**	**43478455**	**43478456**	**7**	**43478475**	**43478476**	**DUP**	**5**	**20**	**HECW1_14**	**HECW1**	**HECW1_14**	**HECW1**	**.**	***FRA7D***
7	44366861	44366862	7	44366921	44366922	DEL	5	60	CAMK2B_intron1	CAMK2B	CAMK2B_intron1	CAMK2B	.	*FRA7D*
7	103516272	103516273	7	103516299	103516300	DEL	5	27	RELN_intron2	RELN	RELN_intron2	RELN	.	*FRA7F*
7	154601421	154601422	7	154601831	154601832	DEL	5	410	DPP6_intron21	DPP6	DPP6_intron21	DPP6	.	
8	6599777	6599778	8	6599797	6599798	DUP	5	20	AGPAT5_intron8	.	AGPAT5_intron8	.	.	
8	56797793	56797794	8	56797813	56797814	DUP	5	20	LYN_intron2	LYN	LYN_intron2	LYN	.	
8	100455975	100455976	8	100461665	100461666	DEL	5	5690	VPS13B_intron29	.	VPS13B_intron29	.	.	
**8**	**116439758**	**116439759**	**7**	**506861**	**506862**	**TRA**		**NA**	**TRPS1_intron5**	**TRPS1**	**.**	**.**		
**9**	**93374497**	**93374498**	**9**	**93374530**	**93374531**	**DEL**	**5**	**33**	**DIRAS2_2**	**.**	**DIRAS2_2**	**.**	**.**	
10	1140984	1140985	10	1140959	1140960	DUP	5	25	WDR37_intron12	.	WDR37_intron12	.	.	
**10**	**44360255**	**44360256**	**10**	**44455497**	**44455498**	**DEL**	**5**	**95242**	**LINC00840_intron3**	**.**	**.**	**.**	**LINC00840-(TRUNC)**	***FRA10G***

Chrom 1	start1	end1	Chrom 2	start2	end2	svtype	Event Score	eventSize	gene1	cancerGene1	gene2	cancerGene2	directFusionCandidates	Fragile sites
10	62684313	62684314	10	62684333	62684334	DUP	5	20	RHOBTB1_intron4	.	RHOBTB1_intron4	.	.	*FRA10C*
**10**	**75548916**	**75548917**	**7**	**100642024**	**100642025**	**TRA**	**3**	**NA**	**ZSWIM8_3**	**.**	**MUC12_5**	**MUC12**	**ZSWIM8-MUC12**	***FRA10G***
10	83693509	83693510	10	83697736	83697737	DEL	5	4227	NRG3_intron4	NRG3	NRG3_intron4	NRG3	.	
**10**	**95089112**	**95089113**	**10**	**95090994**	**95090995**	**DEL**	**5**	**1882**	**MYOF_intron48**	**.**	**MYOF_intron47**	**.**	**MYOF_intron48-MYOF_intron47**	
11	1886893	1886894	11	1886766	1886767	DEL	3	127	LSP1_intron5	.	LSP1_intron5	.	.	
**11**	**6789296**	**6789297**	**11**	**6806935**	**6806936**	**INV**	**2**	**17639**	**OR2AG2_1**	**.**	**OR2AG1_1**	**.**	**OR2AG2-OR2AG1**	
**11**	**17162508**	**17162509**	**11**	**17163785**	**17163786**	**DEL**	**5**	**1277**	**PIK3C2A_intron11**	**PIK3C2A**	**PIK3C2A_intron10**	**PIK3C2A**	**PIK3C2A_intron11-PIK3C2A_intron10**	***FRA11C***
11	28983561	28983562	11	28983604	28983605	DUP	5	43	RP11-115J23.1_intron4	.	RP11-115J23.1_intron4	.	.	
**11**	**49609140**	**49609141**	**11**	**88769938**	**88769939**	**INV**	**2**	**39160798**	**.**	**.**	**GRM5_intron3**	**.**	**(TRUNC)-GRM5**	
11	107300538	107300539	11	107300516	107300517	DUP	5	22	CWF19L2_intron7	.	CWF19L2_intron7	.	.	
**11**	**133242546**	**133242547**	**11**	**133245297**	**133245298**	**DEL**	**5**	**2751**	**OPCML-IT1_intron0**	**OPCML**	**OPCML-IT1_intron0**	**OPCML**	**.**	
12	11836662	11836663	12	11836708	11836709	DUP	5	46	ETV6_intron2	ETV6	ETV6_intron2	ETV6	.	
12	48330567	48330568	12	48330587	48330588	DUP	5	20	VDR_intron1	VDR	VDR_intron1	VDR	.	
12	70657435	70657436	12	70663571	70663572	DEL	5	6136	CNOT2_intron3	.	CNOT2_intron3	.	.	
12	98540255	98540256	12	98540234	98540235	DUP	5	21	RP11-690J15.1_intron2	.	RP11-690J15.1_intron2	.	.	
12	120932416	120932417	12	120932396	120932397	DUP	5	20	DYNLL1_intron5	.	DYNLL1_intron5	.	.	
**12**	**121768476**	**121768477**	**12**	**121769149**	**121769150**	**DEL**	**5**	**673**	**ANAPC5_8**	**ANAPC5**	**ANAPC5_8**	**ANAPC5**	**.**	
12	123754022	123754023	12	123754049	123754050	DUP	5	27	CDK2AP1_intron3	.	CDK2AP1_intron3	.	.	
14	72794968	72794969	14	72794946	72794947	DUP	5	22	RGS6_intron4	.	RGS6_intron4	.	.	

**14**	**85198206**	**85198207**	**14**	**106500730**	**106500731**	**DEL**	**5**	**21302524**	**.**	**.**	**IGH**	**IGH**	**(TRUNC)-IGH**	
**14**	**89076092**	**89076093**	**14**	**89075709**	**89075710**	**DEL**	**3**	**383**	**ZC3H14_21**	**.**	**ZC3H14_21**	**.**	**.**	
15	50635415	50635416	15	50635435	50635436	DUP	5	20	GABPB1_intron2	.	GABPB1_intron2	.	.	
15	77034164	77034165	15	77034328	77034329	DUP	5	164	SCAPER_intron22	.	SCAPER_intron22	.	.	
16	7229859	7229860	16	7251557	7251558	DEL	5	21698	RBFOX1_intron10	.	RBFOX1_intron10	.	.	
16	7251848	7251849	16	7229582	7229583	DUP	2	22266	RBFOX1_intron10	.	RBFOX1_intron10	.	.	
**Chrom 1**	**start1**	**end1**	**Chrom 2**	**start2**	**end2**	**svtype**	**Event Score**	**eventSize**	**gene1**	**cancerGene1**	**gene2**	**cancerGene2**	**directFusionCandidates**	**Fragile sites**
16	63482243	63482244	16	63482318	63482319	DEL	5	75	RP11-368L12.1_intron3	.	RP11-368L12.1_intron3	.	.	
**16**	**66371496**	**66371497**	**1**	**20037267**	**20037268**	**TRA**	**3**	**NA**	**.**	**.**	**TMCO4_intron16**	**.**	**(TRUNC)-TMCO4**	
16	78333837	78333838	16	78338614	78338615	DEL	5	4777	WWOX_intron18	WWOX	WWOX_intron18	WWOX	.	*FRA16D*
16	89373729	89373730	16	89373708	89373709	DUP	5	21	ANKRD11_intron11	.	ANKRD11_intron11	.	.	
**17**	**20741244**	**20741245**	**17**	**22041117**	**22041118**	**DEL**	**2**	**1299873**	**CCDC144NL_intron6**	**.**	**.**	**.**	**CCDC144NL-(TRUNC)**	
17	31839731	31839732	17	31839752	31839753	DUP	5	21	ASIC2_intron2	.	ASIC2_intron2	.	.	
17	35636710	35636711	17	35636730	35636731	DUP	5	20	ACACA_intron17	ACACA	ACACA_intron17	ACACA	.	
17	38562747	38562748	17	38562830	38562831	DEL	5	83	TOP2A_intron16	TOP2A	TOP2A_intron16	TOP2A	.	
18	20458898	20458899	18	20458873	20458874	DUP	5	25	RBBP8_intron2	RBBP8	RBBP8_intron2	RBBP8	.	
18	30935269	30935270	18	30935289	30935290	DUP	5	20	CCDC178_intron10	CCDC178	CCDC178_intron10	CCDC178	.	*FRA18A*
19	4710107	4710108	19	4710084	4710085	DUP	5	23	DPP9_intron10	.	DPP9_intron10	.	.	
19	5695157	5695158	19	5695180	5695181	DUP	5	23	LONP1_intron15	.	LONP1_intron15	.	.	
**19**	**11665070**	**11665071**	**19**	**11664859**	**11664860**	**DEL**	**3**	**211**	**ELOF1_6**	**.**	**ELOF1_7**	**.**	**ELOF1_6-ELOF1_7**	
19	42424084	42424085	19	42424258	42424259	DEL	5	174	ARHGEF1_intron31	ARHGEF1	ARHGEF1_intron31	ARHGEF1	.	*FRA19A*
20	15336088	15336089	20	15336117	15336118	DEL	5	29	MACROD2_intron16	.	MACROD2_intron16	.	.	*FRA20B*
20	17208410	17208411	20	17208334	17208335	DEL	3	76	PCSK2_intron3	.	PCSK2_intron3	.	.	
20	40906734	40906735	20	40906753	40906754	DEL	5	19	PTPRT_intron14	PTPRT	PTPRT_intron14	PTPRT	.	

21	22903293	22903294	21	22903312	22903313	DUP	5	19	NCAM2_intron23	NCAM2	NCAM2_intron23	NCAM2	.	
21	30787123	30787124	21	30787143	30787144	DUP	5	20	BACH1_intron15	BACH1	BACH1_intron15	BACH1	.	
**22**	**24236763**	**24236764**	**22**	**24236955**	**24236956**	**DEL**	**5**	**192**	**MIF_1,AP000350.4_3**	**.**	**AP000350.4_3,MIF_2**	**.**	**MIF_1-MIF_2**	
**X**	**30851678**	**30851679**	**X**	**30852573**	**30852574**	**DEL**	**5**	**895**	**TAB3_intron11**	**TAB3**	**TAB3_intron10**	**TAB3**	**TAB3_intron11-TAB3_intron10**	***FRAXM***
X	69066490	69066491	X	69066516	69066517	DUP	5	26	EDA_intron10	.	EDA_intron10	.	.	
X	107539642	107539643	X	107539662	107539663	DUP	5	20	COL4A6_intron4	COL4A6	COL4A6_intron4	COL4A6	.	
X	123715318	123715319	X	123715340	123715341	DUP	5	22	TENM1_intron11	.	TENM1_intron11	.	.	
X	151420235	151420236	X	151420257	151420258	DUP	5	22	GABRA3_intron6	.	GABRA3_intron6	.	.	
Y	16945610	16945611	Y	16945633	16945634	DUP	5	23	NLGN4Y_intron12	.	NLGN4Y_intron12	.	.	

Chrom: chromosome, **svtype:** structural variation type, **DUP:** duplication, **DEL:** deletion, **INV:** inversion, **TRA:** translocation.

International Journal of
Molecular Sciences
